# The impacts of task shifting on the management and treatment of malnourished children in Northern Kenya: a cluster-randomized controlled trial

**DOI:** 10.1093/heapol/czae036

**Published:** 2024-06-05

**Authors:** Hermann Pythagore Pierre Donfouet, Tewoldeberhan Daniel, Calistus Wilunda, Elizabeth Mwaniki, James Njiru, Emily Keane, Lily Schofield, Lucy Maina, Edward Kutondo, Olivia Agutu, Peter Okoth, Judith Raburu, Betty Samburu, Bonventure Mwangi, Taddese Alemu Zerfu, Jemimah Wekhomba Khamadi, Pilar Charle Cuellar, Daniel Kavoo, Lydia Karimurio, Charles Matanda, Alex Mutua, Grace Gichohi, Martin Chabi, Patrick Codjia, Saul Guerrero Oteyza, Elizabeth Kimani-Murage

**Affiliations:** The World Bank Health Nutrition and Population Global Practice, 1818 H Street, NW, Washington, DC 20433, United States; UNICEF Kenya, UNICEF Eastern and Southern Africa Regional Office, Nairobi 44145, Kenya; African Population and Health Research Center, APHRC Campus, Kitisuru, Nairobi 10787-00100, Kenya; African Population and Health Research Center, APHRC Campus, Kitisuru, Nairobi 10787-00100, Kenya; Save the Children International, 3rd Floor ABC Place Waiyaki Way, Westlands Box 19423, Nairobi 00202 KNH, Kenya; Save the Children UK, St Vincent House, 30 Orange Street, London WC2H 7HH, United Kingdom; Save the Children UK, St Vincent House, 30 Orange Street, London WC2H 7HH, United Kingdom; UNICEF Kenya, UNICEF Kenya Country Office, Nairobi 44145-00100, Kenya; UNICEF Kenya, UNICEF Kenya Country Office, Nairobi 44145-00100, Kenya; UNICEF Kenya, UNICEF Kenya Country Office, Nairobi 44145-00100, Kenya; UNICEF Kenya, UNICEF Kenya Country Office, Nairobi 44145-00100, Kenya; UNICEF Kenya, UNICEF Kenya Country Office, Nairobi 44145-00100, Kenya; UNICEF Kenya, UNICEF Kenya Country Office, Nairobi 44145-00100, Kenya; African Population and Health Research Center, APHRC Campus, Kitisuru, Nairobi 10787-00100, Kenya; African Population and Health Research Center, APHRC Campus, Kitisuru, Nairobi 10787-00100, Kenya; International Food Policy Research Institute, Addis Ababa 5689, Ethiopia; Action Against Hunger-Kenya, Nairobi 39900-00623, Kenya; Action Against Hunger, 6 Mitre Passage, London SE10 0ER, United Kingdom; Ministry of Health, Nairobi 30016-00100, Kenya; Ministry of Health, Nairobi 30016-00100, Kenya; Ministry of Health, Nairobi 30016-00100, Kenya; Ministry of Health, Nairobi 30016-00100, Kenya; Ministry of Health, Nairobi 30016-00100, Kenya; World Health Organization, U-Block, Third floor, United Nations Office, Nairobi 45335, Kenya; UNICEF Kenya, UNICEF Kenya Country Office, Nairobi 44145-00100, Kenya; UNICEF Headquarters, Maiden Lane, New York, NY 10038, United States; African Population and Health Research Center, APHRC Campus, Kitisuru, Nairobi 10787-00100, Kenya

**Keywords:** Task shifting, community health volunteers, non-inferiority cluster-randomized controlled trial, child malnutrition

## Abstract

Treating children with acute malnutrition can be challenging, particularly regarding access to healthcare facilities during treatment. Task shifting, a strategy of transferring specific tasks to health workers with shorter training and fewer qualifications, is being considered as an effective approach to enhancing health outcomes in primary healthcare. This study aimed to assess the effectiveness of integrating the treatment of acute malnutrition by community health volunteers into integrated community case management in two sub-counties in northern Kenya (Loima and Isiolo). We conducted a two-arm non-inferiority cluster-randomized controlled trial across 20 community health units. Participants were children aged 6–59 months with uncomplicated acute malnutrition. In the intervention group, community health volunteers used simplified tools and protocols to identify and treat eligible children at home and provided the usual integrated community case management package. In the control group, community health volunteers provided the usual integrated community case management package only (screening and referral of the malnourished children to the health facilities). The primary outcome was recovery (MUAC ≥12.5 cm for 2 consecutive weeks). Results show that children in the intervention group were more likely to recover than those in the control group [73 vs 50; risk difference (RD) = 26% (95% CI 12 to 40) and risk ratio (RR) = 2 (95% CI 1.2 to 1.9)]. The probability of defaulting was lower in the intervention group than in the control group: RD = −21% (95% CI −31 to −10) and RR = 0.3 (95% CI 0.2 to 0.5). The intervention reduced the length of stay by about 13 days, although this was not statistically significant and varied substantially by sub-county. Integrating the treatment of acute malnutrition by community health volunteers into the integrated community case management programme led to better malnutrition treatment outcomes. There is a need to integrate acute malnutrition treatment into integrated community case management and review policies to allow community health volunteers to treat uncomplicated acute malnutrition.

Key messages

- Addressing acute malnutrition in children can pose difficulties, especially concerning the accessibility of healthcare facilities throughout the treatment process. Task shifting, a strategy involving the delegation of particular responsibilities to health workers with less training and reduced qualifications, is being explored as a viable method to improve health outcomes within primary healthcare.
- This study’s primary objective was to assess the effectiveness of task shifting, specifically employing community health volunteers, to treat malnutrition in two sub-counties of northern Kenya.
- The study seeks to generate evidence that can inform policy and strategy for malnutrition treatment in the region.
- The findings indicate that using community health volunteers to treat malnourished children led to improved treatment outcomes compared to the standard approach.

## Introduction

Despite significant advancements in child survival over the last 30 years, child mortality remains a pressing global concern. In 2019, the number of children under the age of 5 years who died worldwide reached 5.2 million, with sub-Saharan Africa accounting for 53% of these deaths (UNICEF, 2020a). In the same year, 47 million children under 5 years were acutely malnourished; 69% of them in Asia and 27% in Africa (UNICEF, 2020b). The situation is not much different in the East African Community (EAC) countries, as these countries still have large gaps in child mortality and health financing indicators (Table 1). In Kenya, for every 1000 live births, 39 children under the age of 5 years die, and for every 1000 live births, 19 neonates die. The sustainable development goals (SDGs) for child mortality indicators may be more challenging to achieve if there are not enough health professionals. The number of doctors per 10 000 people in Burundi, the Democratic Republic of the Congo, Kenya, Rwanda, South Sudan, Tanzania and Uganda was 1, 4, 1, 2, 0.4, 0.5 and 2, respectively. The same applies for midwives and nurses. To achieve Universal Health Coverage (UHC) service coverage targets (70% of UHC service coverage index), these countries require approximately 134 health workers (a combination of 13 cadres) per 10 000 inhabitants, or a combination of 8 doctors per 10 000 population, and 58.64 nurses and midwives per 10 000 inhabitants (Ahmat *et al*., 2022). This shortage of medical personnel could be alleviated if these countries made significant investments in the health sector. However, according to recent estimates, none of these countries devote 15% of their budget to health, and domestic general government health spending as a percentage of gross domestic product is <5% (Mcintyre *et al*., 2017). This low level of health financing in the East African Community countries could hamper the achievement of SDG 2.2, which calls for the eradication of all types of malnutrition by 2030, including the accomplishment of the internationally agreed-upon targets for stunting and wasting in children under the age of 5 years by 2025.

**Table 1.** East African Community economic and health indicators

				New-born and child mortality indicators			Health financing indicators			Human resources for health indicators	
Country Name	Time	Population	GNI per capita, Atlas method (current US$)	Mortality rate, neonatal (per 1000 live births)	Mortality rate, under-5 (per 1000 live births)	Mortality rate, infant (per 1000 live births)	Out-of-pocket expenditure (% of current health expenditure)	Domestic general government health expenditure (% of general government expenditure)^a^	Domestic general government health expenditure (% of GDP)^b^	Medical doctors (per 10 000)	Nursing and midwifery personnel (per 10 000)
Burundi	2019	11 530 577 11 874 838	230	21.3	56.7	40	30.74	8.31	2.39	0.64 (2021)	7.58 (2021)
Congo, Dem. Rep.	2019	89 906 890	510	27.5	84.4	65.9	38.50	4.36	0.56	3.62 (2018)	10.71 (2018)
Kenya	2019	50 951 450	1890	19.4	39.4	29.7	24.30	8.23	2.01	0.64 (2021)	11.99 (2018)
Rwanda	2019	12 835 028	810	18.3	42.1	31.2	12.13	8.88	2.50	1.16(2019)	9.33 (2019)
South Sudan	2019	10 447 666	NA	39.8	98.7	63.8	22.65	2.11	0.77	0.40 (2018)	3.58 (2018)
Tanzania	2019	59 872 579	1040	20.8	50.4	35.9	21.91	9.44	1.57	0.50 (2018)	5.67 (2018)
Uganda	2019	42 949 080	810	20	45.6	33.2	38.26	3.14	0.57	1.58 (2020)	16.86 (2020)

The data for human resources for health are from the World Health Organization’s Global Health Workforce Statistics. The remaining information comes from the World Bank Development Indicators. We used the most recent data available. The GNI and GDP stand for the gross national income and gross domestic income, respectively. The most recent years for human resources for health are in parentheses. Neonatal mortality rate is the number of neonates dying before reaching 28 days of age, per 1000 live births in a given year. Neonatal mortality rate is the number of neonates dying before reaching 28 days of age, per 1000 live births in a given year. Under-5 mortality rate is the probability per 1000 that a new-born baby will die before reaching age 5 years, if subject to age-specific mortality rates of the specified year. Infant mortality rate is the number of infants dying before reaching one year of age, per 1000 live births in a given year. For the child mortality indicators, the targets for the SDGs are: all countries aim at reducing the neonatal mortality rate to at least 12 per 1000 live births by 2030, and under-5 mortality rate to at least 25 per 1000 live births by the same year. At the time of writing this paper, the Federal Republic of Somalia was not a member of the East African Community. It became a full member on the 4th March, 2024. ^a^All these countries are falling well short of the Abuja objective, which calls for allocating at least 15% of public funds to the health sector. ^b^All these countries are not allocating >5% of their GDP on health.

Acute malnutrition in children, caused by inadequate nutrient intake and/or disease, weakens the immune system, increasing the risk of morbidity and mortality from common childhood illnesses, such as diarrhoea, pneumonia and malaria, and causes long-term developmental delays in survivors. Children suffering from acute malnutrition require immediate treatment and care to survive and thrive. Task shifting may provide a unique opportunity to engage and reach poor and difficult-to-reach populations, resulting in long-term reductions in inequities.

Task shifting was initially developed as a response strategy in the context of HIV and AIDS treatment to strengthen and expand the limited health workforce available to manage the pandemic. This strategy was developed to expand the capacity of health personnel by adding new cadres of workers (WHO, 2008). This strategy has been implemented in the delivery of health services in a variety of settings and for a variety of targeted health outcomes. Task shifting has been also used to care for people with chronic illnesses, prescribe medications and provide health education (Leong *et al*., 2021), and to broaden access to healthcare services, such as antiretroviral therapy and community drug distributions (Harries *et al*., 2006). It has been examined in the provision of mental health services, demonstrating the importance of provider education, supervision and partnerships with local communities in implementation (Hoeft *et al*., 2018). In middle- and low-income countries, task shifting is effective in the management of hypertension (Maria *et al*., 2021). Furthermore, task shifting is cost-effective (Seidman and Atun, 2017), and motivating when lower cadre workers take on more complex tasks and roles, retaining workers in rural settings while improving coverage and access to health services (Munga *et al*., 2012). Although task-shifting has been shown to scale-up treatment (Okyere *et al*., 2017), research indicates that it has complicated clinical procedures, compromised the quality of care, over-burdened those to whom tasks are shifted (Dlamini-Simelane and Moyer, 2017). Less skilled workers run the danger of being overworked or dealing with an uneven workload without receiving adequate compensation (Baine *et al*., 2018). Experience from Tanzania indicate that due to the nature of task-shifting, it is not always clear how the additional tasks support career advancement and promotions for lower cadre staff (Munga *et al*., 2012).

In the case of treating malnourished children in Kenya, task shifting entails assigning uncomplicated malnutrition cases to community health volunteers for treatment, and it has the potential to improve malnourished children’s health outcomes while also increasing the health system’s efficiency. However, there is a lack of solid evidence on the impact of integrating the treatment of acute malnutrition by community health volunteers into the Integrated Community Case Management (iCCM) programme in Kenya. The iCCM is a strategy for managing common childhood illnesses, such as pneumonia, diarrhoea and malaria, at the community level by trained, equipped and supervised community health workers/volunteers (WHO, UNICEF, 2012). The iCCM package also includes the identification and referral of malnourished children to health facilities for either outpatient or inpatient treatment and follow-up, referred to as the community management of acute malnutrition (WHO, 2007). The community management of acute malnutrition has been effective in achieving Sphere standards for malnutrition treatment outcomes (UNICEF, 2013). In areas with significant barriers to accessing health facilities and an existing iCCM programme, integrating community management of acute malnutrition into iCCM can bring malnutrition treatment services closer to children and make optimal use of available resources. In 2014, the prevalence of acute malnutrition among children <5 years in Kenya was 4% (KNBS, 2014). However, this average statistic masks stark regional disparities. Of the 47 counties, 7—all in arid and semi-arid areas—had a prevalence of >10%, with Turkana having the highest prevalence of 23% (KNBS, 2014). The role of community health volunteers in malnutrition management in the country is still limited to screening and referral of malnutrition cases, community mobilization and awareness-raising.

Studies from Asia (Kate Sadler *et al*., 2011a) and Africa (Alvarez Morán *et al*., 2018) suggest that children with severe acute malnutrition (SAM) treated at home by trained, equipped and supervised community-based lay health workers, hereafter referred to as community health volunteers, may achieve better discharge outcomes than those treated in health facilities by professional health workers. Additionally, community health volunteers may increase coverage of acute malnutrition treatment (Amthor *et al*., 2009) and provide care that is of good quality (Puett *et al*., 2013a; Alvarez Morán *et al*., 2018) and cost-effective (Puett *et al*., 2013b; Rogers *et al*., 2018). However, previous studies on the effectiveness of this approach focused on severe acute malnutrition or used a quasi-experimental design (Alvarez Morán *et al*., 2018; Kate Sadler *et al*., 2011b) .

The present study aimed to assess the effectiveness of integrating treatment of acute malnutrition by community health volunteers into iCCM in two sub-counties in northern Kenya to inform malnutrition treatment policy and strategy.

## Materials and methods

### Settings

The study sites were identified based on pre-set criteria including high prevalence of acute malnutrition, long distances from households to health facilities, existence of functional community health units with community health volunteers that have been trained on the basics of the community health strategy, presence of a supportive county government with clear plans for rolling out the community health strategy, existence of a functional supply chain management system for ready-to-use therapeutic food (RUTF) and ready-to-use supplementary food (RUSF), and existence of community health volunteers trained in iCCM. Other criteria were the presence of implementing non-governmental organization (NGO) partners, relative safety and ease of accessibility for implementing organizations and research team. In Kenya, community health services are implemented through community health units. The community health service is the first level of the health system and its workforce in a community health unit includes a community health committee, a community health assistant or a community health officer, and community health volunteers (Ministry of Health, 2020). One community health assistant/community health assistant oversees 10 community health volunteers who oversee up to 5000 people (500–1000 households) while the CHC is the unit’s governing body. The community health assistant/community health assistant are formal employees of the county Ministry of Health.

Turkana and Isiolo counties are characterized by recurrent droughts, hot and dry climate with low and erratic rainfall patterns, and food insecurity. With a total area of 77 000 km^2^ and divided into seven sub-counties, namely Turkana Central, Loima, Turkana South, Turkana East, Turkana North, Kibish and Turkana West, Turkana County is the largest of Kenya’s 47 counties. Located in the northwestern part of Kenya and with a population of 855 399 residents according to the 2009 Kenya Population and Housing Census, Turkana has the highest poverty rate in Kenya at 94% and much worse measures of human development and service coverage than the rest of the nation because of its harsh environment and historical underdevelopment. Only 18% of people can read and write, and only 9% of roads are paved with asphalt. Only one out of every five homes have access to better sanitation (KNBS, 2014). According to a Standardized Monitoring Assessment for Relief and Transition (SMART) survey (SMART, 2015b) conducted in June 2015 covering all the livelihood zones in Turkana County (pastoral, agro-pastoral and formal employment/business/petty trade), about one in four children in the county is acutely malnourished.

Isiolo County, which is in the north-eastern region of Kenya, is bordered to the north by Marsabit County, the west by Samburu and Laikipia Counties, the south by Tana River and Kitui Counties, the east by Wajir County, the southeast by Garissa County, and the southwest by Meru and Tharaka Nithi Counties. The county had an estimated population of 143 294 and a total area of about 25 700 km^2^, with 31 163 children under the age of 59 months being the majority (KNBS, 2009). The three sub-counties that make up this county are Isiolo, Garbatulla and Merti. The county is characterized by recurrent droughts, hot and dry climate with low and erratic rainfall patterns and is prone to food insecurity predisposing vulnerable groups, such as children aged 6–59 months to malnutrition. The county has poor child nutritional indicators. The SMART survey (SMART, 2015a) reported a global acute malnutrition prevalence of 4% (95% CI 2–6) and SAM prevalence of 0.4% (95% CI 0.1–2) based on the mid-upper arm circumference (MUAC).

### Design and intervention

We conducted a two-arm parallel groups, non-inferiority cluster-randomized controlled trial (RCT) in Loima sub-County in Turkana County, and Isiolo sub-County in Isiolo County, northern Kenya. A non-inferiority trial’s goal is to demonstrate that the intervention (experimental treatment) does not fall below a predetermined non-inferiority margin when compared to the control (D’Agostino *et al*., 2003).

A pre- RCT was conducted from January to September 2018 to establish the baseline situation and to inform the design of the RCT, which was conducted from January to September 2019. Children aged 6–59 months were included in the study if they had moderate acute malnutrition (MAM) (MUAC 11.5 to 12.4 cm) or SAM (MUAC < 11.5 cm or presence of bilateral pitting oedema) without medical complications. Children whose caregivers, usually mothers, were not willing to participate in the study were excluded, while those with MUAC < 9.0 cm were also excluded and referred directly to the health facility. Details of the study are available in the published protocol Kimani-Murage *et al*. (2019).

Before the intervention, the implementing NGOs in collaboration with the Kenyan Ministry of Health at the national and county levels trained community health volunteers in the intervention group on the community management of acute malnutrition. Officers from the Ministry of Health trained trainers who in turn trained 70 community health volunteers in Isiolo and 61 CHVs in Loima for 5 days. The training included both theory and practice and focused on using simplified tools and protocols initially developed, tested and used for management of acute malnutrition by low-literate community health volunteers in a similar context (Kozuki *et al*., 2020). The tools, which consisted of a modified MUAC tape, a modified weighing scale, a dosage calculator and treatment registers, were adapted to the local context. The implementing partners also conducted a 3-day refresher training on iCCM for community health volunteers in both the intervention and control groups. In both the intervention and control groups, community health volunteers sought for and screened children with acute malnutrition in the community and enrolled all those who were eligible in the study. In the control group, community health volunteers referred malnourished children to health facilities for treatment by professional health workers according to the national guidelines. The estimated number of malnourished children was randomly chosen from the community health volunteers’ registers and followed up in each of the two arms as per the national guidelines. Treatment outcomes were extracted from community health volunteers’ registers in the intervention group and from the health facility registers in the control group. This is because in the intervention group, community health volunteers treated malnourished children in the community but in the control group children were treated in health facilities. Enumerators and community health volunteers both participated in the two arms for data collection. These enumerators collaborated closely with community health volunteers and other healthcare personnel within the health facility. These enumerators were uniformed throughout the trial and had received training in data collection. The activities of enumerators were monitored by study supervisors to ensure the quality of data collection. The team leader double-checked the documentation.

Details of the treatment protocol in the intervention group are shown in the Supplementary Appendix Table S1. Screening in this group involved examining children for danger signs and illness including cough/cold, diarrhoea, dehydration and fever, malaria testing using a rapid diagnostic test (RDT), weighing the child and taking MUAC measurements using a special five colour-band tape to classify children into three groups: normal (green: MUAC >12.5 cm), moderate acute malnutrition (yellow: MUAC 11.5 cm and 12.5 cm) or severe acute malnutrition (pink, brown or bright red: MUAC of < 11.5 cm). Children with MUAC < 9.0 cm (bright red), bilateral pitting oedema and/or <4 kg, with no appetite, sick (according to any of the signs in Supplementary Appendix Table S1) or acutely malnourished but younger than 6 months were referred to health facilities. Those with a good appetite and no medical complications were recruited in the study for community-based treatment by CHVs.

Children with SAM received RUTF according to their weight (Supplementary Appendix Table S1) for daily intake for 7 days (until the next visit). Information on how to feed the child was given to the caregiver. They also received the first dose of amoxycillin dispersible tablets on the spot and twice for 7 days before the next visit. If the malaria test was positive, community health volunteers gave one dose of ACT on the spot and a twice daily for 3 days prescription, with an explanation on how to administer to the caregiver. Children with MAM received RUSF for 14 days until the next visit. Albendazole and folic acid (appropriate for weight) were given on the spot and further dosage was explained to the caregiver. Mothers were advised on the follow-up, breastfeeding and diversification of feeding in addition to RUSF.

During follow-up visits, community health volunteers in the intervention arm sought for general danger signs including cough, fever or diarrhoea and any other complaints on the child according to the standardized treatment and follow-up protocol. Anthropometric measurements (weight and MUAC) were taken to monitor any changes in the child’s nutritional status. The community health volunteers also examined children for oedema and conducted an appetite test. Children with SAM with failed appetite test, 4 consecutive weeks with dark red MUAC, 4 consecutive weeks with pink MUAC, any MUAC regression, 3 consecutive weeks with no weight gain or any weight regression were referred to the health facility. The criteria for referral among children with MAM included failed appetite test, any MUAC regression, 3 consecutive weeks with no weight gain, or any weight regression. Children with SAM received RUTF for use in the next 7 days and if RDT was positive, ACT was provided on the spot and twice daily for 3 days. Albendazole was given during the second visit if the child was aged more than 12 months.

Children with MAM received folic acid and continued with RUSF for the next 14 days. Providing a diverse diet breastfeeding, handwashing and use of bed net were emphasized at this point. Furthermore, during follow-up, all children with SAM were visited (or caregivers visited the community health volunteers) weekly by CHVs for MUAC and weight measurement and refilling of RUTF as per the dosage scale. A maximum of eight weekly visits was conducted if the child was progressing well, otherwise the child was referred to the nearby health facility in case of non-response to treatment. Children with MAM were visited (or caregivers visited the community health volunteers) once every 2 weeks for MUAC measurement and replenishment of RUSF (one sachet per day for 16 weeks for a maximum of eight visits if the child was progressing well, otherwise the child was referred).

### Randomization and masking

Community health units, defined as health service structures with a defined geographical area and assigned to a health facility, comprised clusters and were the unit of randomization to minimize the risk of contamination, and to align with community health volunteers’ territory. A total of 10 community health units were included in each sub-county. Given the small number of clusters, paired-matched randomization (with a 1:1 allocation ratio) was used to ensure treatment groups were balanced and to increase study power (Balzer *et al*., 2015). Matching was based on the cluster-level distribution of maternal and household socio-demographic characteristics (age, income and education) collected during the pre-RCT. Randomization was performed by a statistician who was not part of the research team. Due to the nature of the intervention, masking of participants, health facility staff and community health volunteers was not possible. Outcome data collection and statistical analyses were also not masked.

### Outcomes

The primary study outcome was recovery while the secondary study outcomes were default, non-response, death, length of stay and weight gain. The outcomes were defined in a standardized way in the control and intervention groups according to the Kenyan National Guidelines for Integrated Management of Malnutrition. Recovery was defined as two consecutive green readings on the colour-coded MUAC, corresponding to ≥ 12.5 cm. Defaulters were children who were absent on three consecutive visits. Non-responders were children who did not meet the discharge criteria after a maximum treatment period of 16 weeks or with deteriorating MUAC. Length of stay was defined as the number of days from treatment initiation to recovery among recovered children. Weight gain was defined as weight change in gram per kilogram per day from entry to exit from the study among recovered children.

Outcomes were evaluated weekly for children with SAM and every 2 weeks for children with MAM by community health volunteers in the intervention arm or healthcare workers in health facilities through MUAC and weight measurement, physical assessment and review of treatment registers. Table 2 provides the definitions of the different outcomes following the Kenyan national guidelines.

**Table 2.** Definition of performance indicators

Performance indicator	Definition
Recovery rate	Proportion of children discharged cured of total discharged. The respondent is cured if there are two consecutive green readings on the colour-coded MUAC.
Defaulter rate	Proportion of children recorded as absent for 3 consecutive weeks, out of the total discharged.
Non-response rate	Proportion of children followed up who do not meet the discharge criteria after the treatment period of 16 weeks, out of the total discharged.
Average length of stay	Number of days from treatment initiation to exit from the study due to recovery, death, default or non-response among the cured children.
Average weight gain	Weight change in *gram* per *kilogram* per day from treatment initiation to exit from the study among cured children.

Total number of discharged = cured + died + defaulted + non-response.

### Statistical analysis

To test whether task shifting for the treatment of children with acute malnutrition is not worse than the standard of care by a non-inferiority margin, we employed non-inferiority RCT. We had in-depth discussions with nutrition and public health experts, and it was decided that a non-inferiority RCT design was appropriate because task shifting was thought to be either slightly more or less effective than the standard care for treating children with acute malnutrition. Furthermore, it was mentioned that the task-shifting policy would be more practical and cost-effective for treating malnourished children in Turkana and Isiolo, even though it may not be superior to conventional care. Specifically, it was argued that providing community health volunteers with substantial support in the form of training, supervision, supplies and logistics could lessen challenges (such as long travel times to medical facilities, serious shortages of healthcare workers and high opportunity costs for mothers who needed to care for the malnourished child) of accessing treatment for malnutrition in remote villages in Turkana and Isiolo.

The choice of the non-inferiority margin is crucial. It was difficult to estimate the non-inferiority margin based on earlier RCTs because there had not yet been any RCTs of task shifting for treating malnourished children at the time of the investigation. The historical data-based fixed-margin method (Althunian *et al*., 2017) was therefore not an option. We rather relied on the opinion of experts to ascertain the maximum loss of the effect that they are willing to accept at the expense of gaining other benefits that are allegedly provided by the intervention. As a result, a 15% non-inferiority margin was selected. The literature suggests choosing a non-inferiority margin based on expert opinion (Schumi and Wittes, 2011; Dunn *et al*., 2018).

The sample size was calculated based on the primary outcome of recovery from acute malnutrition, which was estimated to be 75% based on routine data from the study sub-counties. The study assumed that treatment of children with acute malnutrition would be non-inferior compared to the facility-based treatment by professional health workers. Using the sample size formula for non-inferiority cluster-RCTs (Rutterford *et al*., 2015) and assuming an intra-cluster correlation coefficient of 0.005, one-sided test with a 95% confidence interval, 80% power, a cluster size of 24 participants, an attrition rate of 5% and a non-inferiority limit of 15%, the minimum required sample size for each arm was 120 children per study site, i.e. a total of 240 children in 10 community health units in Loima, and 240 children in 10 community health units in Isiolo.

The analysis was by intention-to-treat. In the primary analysis, children in the intervention group who were transferred to health facilities according to the intervention protocol and their outcomes could not be ascertained because they could not be identified in the treatment registers were addressed in the sensitivity analysis using a parametric approach and non-parametric approach. For the parametric approach, we use the inverse probability weighting (IPW). To mitigate the potential bias caused by missing data, we calculated inverse probability weights by taking the reciprocal of the estimated probability of being assessed at follow-up, as predicted by all baseline characteristics. These weights were then applied in all regression analyses to evaluate the impact of the interventions. By incorporating these weights, our analyses aimed to accurately adjust for the likelihood of missing data and provide more precise estimates of the intervention effects. Similarly, in the non-parametric methods, we used the Lee’s bound (Lee, 2009) that allows us to establish boundaries for the treatment effect that are resilient against differential attrition.

Due to the limited number of clusters in this study, appropriate methods for analysing few clusters (Hayes and Moulton, 2017) were employed. This involved calculating cluster-level risk ratios and risk differences for binary outcomes (recovery, default and non-response) or mean differences for continuous outcomes (length of stay and weight gain) and pooling the effect estimates using inverse variance-weighted random effects meta-analysis to account for unequal cluster sizes and heterogeneity across clusters, which was measured using the *I*^2^ statistic. The cluster-level risk difference (RD) and risk ratio (RR) were computed using a Stata user-written program *csti*, while the mean differences were obtained using linear regression.

Non-inferiority was evaluated by comparing the lower bound of the 95% CI for the effect of the intervention on recovery with the pre-specified non-inferiority margin of −15%. The effect of the intervention on death was not assessed because of zero counts. Statistical analysis was done with Stata version 16.1 (StataCorp LP, College Station, TX, USA). The trial was pre-registered in the Pan African Clinical Trials Registry (PACTR201811870943127). The study protocol and tools were approved by the African Medical and Research Foundation (AMREF) Health Africa Ethical and Scientific Review Committee (number P416/2017).

## Results

**Figure 1.**
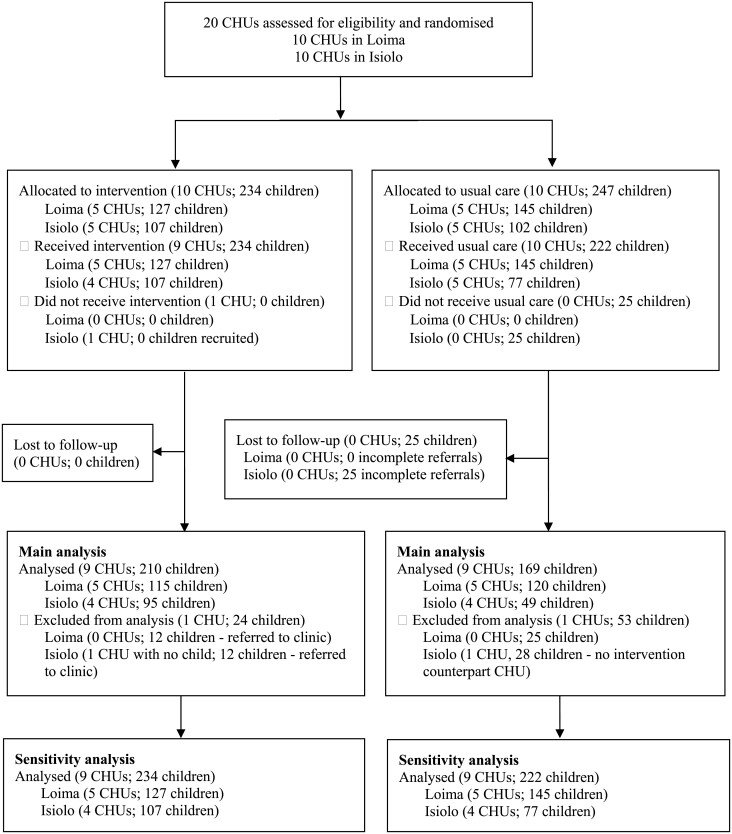
Cluster RCT profile

Notes: This is a flowchart of a cluster randomized controlled trial (RCT) profile. In each sub-county, the chart begins with the identification and assessment of eligible clusters, followed by random allocation into intervention and control groups. Each group then proceeds through the trial with participants assessed for outcomes. CHU=community health units.

A total of 481 children in 20 community health units (10 community health units with 272 children in Loima and 10 community health units with 209 children in Isiolo) were recruited in the RCT (Figure 1). Ten community health units with 234 children were randomly allocated to the intervention group and 10 community health units with 247 children were randomly allocated to the control group. All the children enrolled in the intervention group in both sites started treatment. However, in the control group (usual care), 100% (145/145) and 75% (77/102) of the children started treatment in Loima and Isiolo, respectively. Non-initiation of treatment (no receipt of usual care) in the control group was due to failure to visit health facilities after referral (incomplete referral). All incomplete referrals (*n* = 25) were included in the analysis as randomized. In the intervention group, 81 children (52 in Loima and 29 in Isiolo) were referred by CHVs to health facilities for either weight deterioration, weight stagnation or illness according to the intervention protocol. Out of these, we identified 57 (40 in Loima and 17 in Isiolo) in health facility registers, ascertained their treatment outcomes and included them in the analysis as randomized. We faced difficulties in locating 24 out of 81 children (12 in Loima and 12 in Isiolo) in the health facility registers. Despite this challenge, we conducted a sensitivity analysis to address the missing data. This analysis utilized the IPW and Lee’s bounds to provide a thorough evaluation of the potential impacts, despite the missing information. We excluded one matched pair of community health units in Isiolo because community health volunteers did not recruit any malnourished child in one of the pair members. The main analysis included 9 community health units with 210 children in the intervention group and 9 clusters with 169 children in the control group, while the sensitivity analysis consisted of 9 community health units with 234 children in the intervention group and 9 clusters with 222 children in the control group.

The intervention and control groups were comparable based on maternal and child background characteristics (Table 3). However, overall, the proportion of female children was higher in the intervention group (67%) than in the control group (59%). Moreover, children in the intervention group were more likely to have been fully vaccinated (40%) than those in the control group (29%). About 89% of children in both study arms had MAM.

**Table 3.** Baseline characteristics of participants

	Loima		Isiolo		Total	
Characteristics	Control (*n* = 145)	Intervention (*n* = 127)	Control (*n* = 102)	Intervention (*n* = 107)	Control (*n* = 247)	Intervention (*n* = 234)
Caregivers						
Age, years	29.6 ± 7.7	31.6 ± 9.4	29.8 ± 8.1	29.0 ± 7.8	29.4 ± 8.4	30.3 ± 9.0
Household size	7.5 ± 1.9	7.3 ± 2.1	6.9 ± 1.6	6.2 ± 2.1	7.1 ± 1.3	6.8 ± 1.5
Married/co-habiting	135 (93.1)	126 (99.2)	81 (79.4)	93 (86.9)	216 (87.4)	219 (93.6)
Education						
No education	111 (76.6)	108 (85.0)	75 (73.5)	71 (66.4)	186 (75.3)	179 (76.5)
At least primary	34 (23.4)	19 (15.0)	27 (26.5)	36 (33.6)	61 (24.7)	55 (23.5)
Occupation						
Unemployed	8 (5.5)	6 (4.7)	40 (39.2)	47 (43.9)	48 (19.4)	53 (22.6)
Employed/self-employed	137 (94.5)	121 (95.3)	62 (60.8)	60 (56.1)	199 (80.6)	181 (77.4)
Wealth index^a^						
Lowest	30 (20.7)	29 (22.8)	13 (12.7)	20 (18.7)	43 (17.4)	49 (20.9)
Second	45 (31.0)	22 (17.3)	25 (24.5)	25 (23.4)	70 (28.3)	47 (20.1)
Middle	38 (26.2)	45 (35.4)	21 (20.6)	19 (17.8)	59 (23.9)	64 (27.4)
Fourth	18 (12.4)	18 (14.2)	23 (22.5)	27 (25.2)	41 (16.6)	45 (19.2)
Highest	14 (9.7)	13 (10.2)	20 (19.6)	16 (15.0)	34 (13.8)	29 (12.4)
Children						
Age, months	21.9 ± 12.4	22.3 ± 13.6	19.9 ± 11.6	18.1 ± 11.4	21.1 ± 12.1	19.8 ± 12.6
Female	82 (56.6)	94 (74.0)	64 (62.7)	62 (57.9)	146 (59.1)	156 (66.7)
Nutritional status						
MAM	127 (87.6)	116 (91.3)	92 (90.2)	92 (86.0)	219 (88.7)	208 (88.9)
SAM	18 (12.4)	11 (8.7)	10 (9.8)	15 (14.0)	28 (11.3)	26 (11.1)
Vaccination						
BCG	36 (24.8)	50 (39.4)	53 (52.0)	67 (62.6)	89 (36.0)	117 (50.0)
Pentavalent	36 (24.8)	49 (38.6)	52 (51.0)	67 (62.6)	88 (35.6)	116 (49.6)
Polio	35 (24.1)	48 (37.8)	50 (49.0)	69 (64.5)	85 (34.4)	117 (50.0)
Measles	31 (21.4)	39 (30.7)	43 (42.2)	61 (57.0)	74 (30.0)	100 (42.7)
Pneumococcal	36 (24.8)	50 (39.4)	50 (49.0)	66 (61.7)	86 (34.8)	116 (49.6)
Fully vaccinated	30 (20.7)	35 (27.6)	41 (40.2)	59 (55.1)	71 (28.7)	94 (40.2)

Data are mean ± SD or *n* (%). MAM: moderate acute malnutrition; SAM: Severe acute malnutrition; BCG: Bacillus Calmette–Guérin. ^a^Constructed using principal component analysis based on ownership of household assets, type of housing material and access to utilities.

Table 4 shows the results of the effectiveness of the intervention on treatment outcomes. Children in the intervention group were more likely to recover than those in the control group [73 vs 50; risk difference (RD) = 26% (95% CI 12 to 40) and risk ratio (RR) = 2 (95% CI 1.2 to 1.9)]. The effects were slightly stronger in Isiolo than in Loima with little heterogeneity across cluster pairs (Supplementary Appendix Table S2). Similarly, results in Table 4 suggests that the probability of defaulting was lower in the intervention group (9%) than in the control group (28%); RD = −21% (95% CI −31 to −10) and RR = 0.3 (95% CI 0.2 to 0.5) with moderate to substantial heterogeneity across cluster pairs (Supplementary Appendix Table S2). The intervention reduced the length of stay by about 31 days in Isiolo [mean difference (MD) =  −31 (95% CI −50 to −11)] but had no significant effect in Loima and or the two sites combined. Overall, weight gain was higher by about 1 g/kg/day in the intervention group than in the control group [MD = 0.8 (95% CI −1 to 3)], but not significant. The intervention had no effect in non-response.

**Table 4.** Impacts of treatment of acute malnutrition by community health volunteers (main analysis based on the intention-to-treat analysis)

	Isiolo	Loima	Total
**Recovery**			
Control, *n* (%)	24 (49.0)	61 (50.8)	85 (50.3)
Intervention, *n* (%)	76 (80.0)	78 (67.8)	154 (73.3)
RD (95% CI)	26.7 (9.0, 44.4)	20.4 (3.5, 37.3)	25.7 (11.7, 39.6)
RR (95% CI)	1.51 (1.13, 2.01)	1.42 (1.04, 1.93)	1.53 (1.18, 1.97)
**Default^a^**			
Control, *n* (%)	16 (32.7)	31 (25.9)	47 (27.8)
Intervention, *n* (%)	8 (8.4)	11 (9.6)	19 (9.0)
RD (95% CI)	−22.9 (−39.1, −6.7)	−19.8 (−32.9, −6.8)	−20.8 (−31.3, −10.2)
RR (95% CI)	0.27 (0.12, 0.62)	0.30 (0.15, 0.57)	0.29 (0.17, 0.49)
**Nonresponse^a^**			
Control, *n* (%)	9 (18.4)	27 (22.7)	36 (21.3)
Intervention, *n* (%)	11 (11.6)	26 (22.6)	37 (17.6)
RD (95% CI)	−4.9 (−15.6, 5.7)	−1.2 (−14.5, 12.1)	−5.5 (−16.5, 5.5)
RR (95% CI)	0.71 (0.33, 1.48)	0.95 (0.53, 1.70)	0.75 (0.43, 1.31)
**Length of stay, days^b^**			
Control, mean ± SD	72.7 ± 29.4	52.6 ± 23.1	58.2 ± 26.5
Intervention, mean ± SD	50.5 ± 23.4	50.7 ± 22.7	50.6 ± 22.9
MD (95% CI)	−30.64 (−49.89, −11.38)	−2.17 (−24.82, 20.47)	−12.67 (−30.92, 5.48)
**Weight gain, g/kg/day ^b^**			
Control, mean ± SD	0.8 ± 1.0	2.0 ± 3.1	1.7 ± 2.1
Intervention, mean ± SD	1.4 ± 3.2	2.7 ± 5.1	2.1 ± 4.3
MD (95% CI)	0.66 (−0.58, 1.91)	0.78 (−2.31, 3.87)	0.75 (−1.07, 2.56)

a In Isiolo, one cluster pair was dropped in estimating the risk ratio because no child had the outcome.

b Among the recovered children. MD: Mean difference.

**Figure 2.**
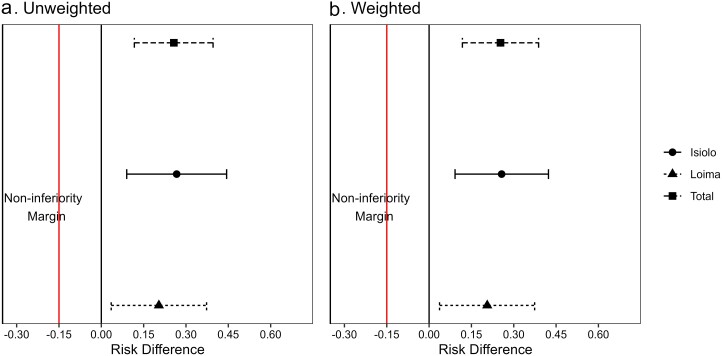
Evaluation of non-inferiority of the intervention based on recovery

The analysis of the lower bound of the 95% CI for the recovery RD compared to the pre-specified non-inferiority margin of −15% indicates that the intervention was non-inferior compared to the standard of care (Figure 2). Specifically, the lower bounds of the 95% CI were above the non-inferiority margin of −15% (−0.15) for recovery, indicating that the new intervention was non-inferior to the standard care (Figure 2). Additionally, since the 95% CI did not include the null value (0), the new intervention can also be considered superior to the standard care. This means that using the community health volunteers to screen and treat malnutrition resulted in a cure rate that was at least as good as (based on the set margin) or better than the cure rate in health facilities.

The error bars represent 95% CIs around the risk difference. This figure reports the 95% CI for the recovery rate based on the intention-to-treat analysis (unweighted) and inverse probability weighting (weighted).

The analysis that accounted for missing outcomes for 24 children produced results consistent with those obtained in the main analysis (Table 5 and Supplementary Appendix Table S3). However, the effect of the intervention on weight gain for the overall sample became significant [RD = 1.0 (95% CI 0.3 to 1.6)] when using a meta-analysis approach (Supplementary Appendix Table S2).

**Table 5.** Impacts of treatment of acute malnutrition by community health volunteers [sensitivity analysis based on the use inverse probability weighting (IPW)]

	Isiolo	Loima	Total
**Recovery**			
Control, *n* (%)	24 (50.4)	61 (50.1)	85 (50.2)
Intervention, *n* (%)	76 (79.9)	78 (67.5)	154 (73.4)
RD (95% CI)	25.7 (9.2, 42.3)	20.6 (3.7, 37.4)	25.3 (11.8, 38.8)
RR (95% CI)	1.48 (1.14, 1.93)	1.42 (1.04, 1.94)	1.52 (1.19, 1.93)
**Default**			
Control, *n* (%)	16 (30.8)	31 (26.7)	47 (28.0)
Intervention, *n* (%)	8 (8.3)	11 (9.7)	19 (9.1)
RD (95% CI)	−21.1 (−37.4, −4.8)	−20.2 (−33.9, −6.4)	−20.7 (−31.6, −9.8)
RR (95% CI)	0.29 (0.13, 0.67)	0.29 (0.15, 0.58)	0.29 (0.17, 0.49)
**Nonresponse**			
Control, *n* (%)	9 (18.9)	27 (22.3)	36 (21.2)
Intervention, *n* (%)	11 (11.7)	26 (22.8)	37 (17.5)
RD (95% CI)	−5.1 (−15.1, 4.9)	−0.7 (−13.8, 12.3)	−5.0 (−15.5, 5.5)
RR (95% CI)	0.71 (0.36, 1.40)	0.97 (0.54, 1.72)	0.77 (0.45, 1.32)
**Length of stay, days^a^**			
Control, mean ± SD	76.3 ± 31.0	52.8 ± 27.9	58.2 ± 26.5
Intervention, mean ± SD	50.0 ± 25.9	49.8 ± 26.1	50.6 ± 22.9
MD (95% CI)	−29.54 (−47.58, −11.49)	−2.69 (−25.56, 20.18)	−13.84 (−31.56, 3.89)
**Weight gain, g/kg/day ^a^**			
Control, mean ± SD	0.6 ± 0.9	1.9 ± 4.1	1.7 ± 2.1
Intervention, mean ± SD	1.3 ± 3.5	2.9 ± 5.8	2.1 ± 4.3
MD (95% CI)	0.59 (−0.59, 1.78)	0.79 (−2.29, 3.82)	0.77 (−0.99, 2.53)

a Among the recovered children. MD: Mean difference.

## Discussions

This cluster-RCT conducted in northern Kenya evaluated the integration of treatment for acute malnutrition by community health volunteers into iCCM. The results showed that compared to the standard of care, the intervention increased the probability of recovery by 26 percentage points (a relative increase of 53%) and reduced the probability of defaulting by 21 percentage points (a relative reduction of 71%). Additionally, children treated by community health volunteers had a shorter length of stay, particularly in Isiolo, and a higher weight gain, particularly in Loima, compared to those treated in health facilities. The intervention had no effect on non-response to treatment. The intervention was found to be non-inferior and superior to the standard of care with respect to the primary outcome.

Our results indicate that the higher probability of recovery in the intervention group was mainly driven by reduced defaulting, rather than non-response to treatment. This suggests that the intervention addressed the barriers that children faced in complying with the full treatment schedule once admitted to the treatment programme. A study in Mali found that SAM treatment by community health volunteers decreased defaulter rates due to improved access, reduced need to travel and proximity of CHVs (López-Ejeda *et al*., 2020). In our study, 10% (25/247) of children referred by CHVs to health facilities in the control group did not go to health facilities as referred, highlighting the limitations of traditional facility-based management of acute malnutrition programmes. A similar problem was observed in a study in Bangladesh, where 53% of caregivers of malnourished children refused hospital referral, although this was for inpatient care (Kate Sadler *et al*., 2011a). This can be attributed to barriers in access to healthcare. We found no significant difference in morbidity (diarrhoea, fever and cough) between the intervention and control arms in both Loima and Isiolo, suggesting that CHVs in the intervention group continued to diagnose and treat children in line with iCCM as those in the control group. The impact of the intervention was slightly stronger in Isiolo compared to Loima. The community health volunteers in Isiolo received a monthly stipend of about US$30 while those in Loima did not receive a stipend. While the provision of monthly stipends to community health volunteers in Isiolo likely boosted their motivation to effectively implement the intervention, further investigation is necessary to explain the variation in the strength of the intervention across the two sub-counties.

This study was overseen by a technical advisory group (TAG) formed within the Ministry of Health to provide strategic and technical advice on issues related to the study design and intervention delivery. The TAG, which included experts from the national and international agencies, was involved in the training of community health volunteers, the simplification of MUAC screening and community health volunteers’ registries, and the management of malnutrition commodities. This governance structure played a key role in the implementation of task shifting. A well-established governance structure has been shown to improve health outcomes (Bokhari *et al*., 2007; Lazarova and Mosca, 2008; Farag *et al*., 2013). The community health strategy provided a supportive framework for operational organization, supervision and mentorship, commodity management and reporting. Implementing this intervention in other settings will require strengthening the capacity of the community health strategy, including the community health information system to meet the monitoring and evaluation needs of the programme.

Our findings are consistent with emerging evidence on the effectiveness of treatment of acute malnutrition by community health volunteers and support the integration of this service in iCCM. A review of published studies and grey literature on operational experiences in treatment of SAM through community health platforms in Africa and Asia found that community health volunteers can identify and treat children with severe acute malnutrition without complications, often achieving recovery rates above the minimum standards and reducing default rates (López‐Ejeda *et al*., 2019). Subsequent studies have supported the conclusions of this review. For example, a quasi-experimental study in northern Tanzania found that children with uncomplicated SAM treated by community health volunteers were more likely to recover (91%) than those treated in health facilities (75%) (Wilunda *et al*., 2021). A recent study in South Sudan, which did not include a comparator, reported a recovery rate of 91% among children treated by community health volunteers (Kozuki *et al*., 2020). However, not all studies have found treatment of malnutrition by community health volunteers to be more effective than facility-based treatment. For instance, a cluster-RCT in Pakistan reported no significant difference in recovery between the children treated by community health volunteers and those treated in health facilities (79% vs 86%) (Hussain *et al*., 2021). The study also found no effect of the intervention on defaulting. In this current study, we observed a higher probability of recovery in the intervention group compared to facility-based care, which can be attributed to the availability of simplified tools and equipment, and the beneficial effects of mentoring, on-the-job training and supervisory structure for the community health volunteers. Higher recovery rate in the intervention group could also be attributed to regular home visits by community health volunteers, which was accompanied by health education on topics such as appropriate use of therapeutic foods, childcare practices, breastfeeding, complementary feeding, food security and hygiene and sanitation. Consistent with results from a quasi-experimental study in Tanzania (Wilunda *et al*., 2021), this study observed no effect of the intervention on length of stay overall. However, contrary to the study in Tanzania (Wilunda *et al*., 2021), weight gain was higher in the intervention group than in the control group.

To the best of our knowledge, this is the first study to evaluate the impact task shifting on the management and treatment of malnourished children using an RCT in resource-limited settings with a high a burden of acute malnutrition. The findings of this study are generalizable to similar settings. However, the study has some limitations. First, in the intervention group, children whose weight deteriorated or stagnated and those who developed an illness were transferred to health facilities according to the intervention protocol, which may have led to an overestimation of the impact of the intervention. However, the sensitivity analysis yielded results similar to those in the main analysis. Second, our study included children with both MAM and SAM, and it was not possible to stratify our results by these conditions because the number of children with SAM was too small. Fourth, this study included few clusters, which might lead to inflation of type I error rates. We conducted cluster-level analysis, as recommended (Leyrat *et al*., 2018), to account for this in sensitivity analysis. The results were similar to those of the main analysis. Fifth, this study was conducted in the pre-COVID-19 pandemic period. However, the findings could be relevant in the context of the COVID-19 pandemic, where access to health services may have been negatively affected.

## Conclusions

This study shows that treatment of acutely malnourished children by the community health volunteers increased the probability of recovery and reduced the probability of default. The intervention also led to an overall increase in weight gain. These findings highlight the importance of integrating treatment of acute malnutrition by the community health volunteers into iCCM and reviewing policies to allow community health volunteers to treat uncomplicated acute malnutrition at home. This task shifting strategy could accelerate access to treatment of acute malnutrition in areas with limited access to health services and contribute towards achieving the goal of the Kenya Health Policy 2014–2030 of attaining the highest possible standard of health for all citizens.

## Supplementary Material

czae036_Supp

## Data Availability

The data underlying this article will be made available on request from the African Population and Health Research Center through the Microdata portal (https://aphrc.org/microdata-portal/) after the paper has been accepted for publication.

## Reflexivity statement

The research was designed to assess the effectiveness of task shifting, specifically employing community health volunteers to treat malnutrition in two sub-counties of northern Kenya. Using a cluster RCT design, our objective was to generate evidence that can inform malnutrition treatment policy and strategy. Our paper embodies inclusivity and diversity in multiple aspects. It is a collaborative effort among several authors affiliated with different organizations across three continents. H.P.P.D. is currently affiliated with the World Bank Group (Washington, D.C.), E.K.-M., C.W., E.M., B.M. and T.A.Z. are associated with the African Population Health Research Center (Nairobi, Kenya). J.N. is associated with Save the Children International (Nairobi, Kenya). E.K., L.S. are affiliated with Save the Children International (United Kingdom, London). T.D., L.M., E.K., O.A., P.O., J.R., B.S., P.C. and S.G.O. are affiliated with UNICEF. J.K.W. is affiliated with Action Against Hunger (Nairobi, Kenya). D.K., L.K., C.M., A.M. and G.G., are affiliated with the Ministry of Health (Nairobi, Kenya). The authors represent a wide range of seniority and experience. They possess extensive expertise in conducting quantitative research in the fields of health policy, health financing and malnutrition, particularly in Africa. E.K.-M., C.W., E.M., B.M., T.D., L.M., E.K., O.A., P.O., J.R., B.S., are from a middle-lower-income country (Kenya). Of the 26 co-authors, 12 are females.

## Disclaimer

The views expressed in this paper are solely those of the authors and do not necessarily reflect the views of any organization or individual mentioned. The leading author is a staff member of the World Bank Group, but the findings, interpretations, and conclusions expressed in this paper should not be attributed in any way to the World Bank, its affiliated organizations, or members of its Board of Executive Directors or the countries they represent. Additionally, some co-authors are from UNICEF, but UNICEF does not accept any responsibility or liability with regard to the views, analysis, or interpretation of the results expressed in this paper. Once again, the findings, interpretations, and conclusions expressed in this paper are solely those of the authors and should not be attributed in any way to UNICEF.
