# Supplementary material for: The impacts of task shifting on the management and treatment of malnourished children in Northern Kenya: a cluster-randomized controlled trial
Source: Health Policy Plan. 2024 Jun 5;39(7):710–21. doi: 10.1093/heapol/czae036 (PMC11308611; doi:10.1093/heapol/czae036)
Supplement: czae036_Supp [file czae036_supp.zip › HPPAUG0511Supplementary-Tables.docx]

**Appendix Table 1: A summary of the intervention protocol**

|  | **SAM** | | **MAM** |
| --- | --- | --- | --- |
| **Inclusion criteria for children to be treated** | Children with brown or pink MUAC (9-<11.5cm) with no complications, between ages 6-59 months | | Children with yellow MUAC (11.5-<12.5cm) with no complications, between ages 6-59 months |
| **Definition of SAM/MAM** | Brown or pink MUAC (9-<11.5cm) | | Yellow MUAC (11.5-<12.5cm) |
| **Definition of cured** | Child is discharged having reached the cure discharge criteria for 2 consecutive weeks:  Green MUAC (≥12.5 cm) | | Child is discharged having reached the cure discharge criteria for 2 consecutive weeks:  Green MUAC (≥12.5 cm) |
| **Definition of non-response** | Child is discharged having not achieved the cured discharge criteria after 16 weeks in treatment | | Child is discharged having not achieved the cured discharge criteria after 16 weeks in treatment |
| **Definition of default** | Child is absent for 3 consecutive visits | | Child is absent for 3 consecutive visits |
| **Follow-up of defaulters** | CHVs conducted a home visit when a child missed a treatment visit; other formal follow up was not planned for defaulters in this study | | |
| **Danger signs which trigger referral** | Identification of an iCCM danger sign:   - Cough 14+ days - Diarrhoea 14+ days - Blood in stool - Fever 7+ days - Convulsions - Unable to eat or drink - Vomits everything - Unconscious - Abnormally sleepy - Chest indrawing - Oedema (swelling of both feet) - Fast breathing (indication of pneumonia, CHVs provided pre-referral treatment only)   OR   - Bilateral oedema - Bright red MUAC (<9cm) | | |
| **Other referral triggers (e.g., stagnant MUAC / weight)** | - Failed appetite test - 4 consecutive weeks with brown MUAC (9-<10.25cm) - 4 consecutive weeks with pink MUAC (10.25-<11.5cm) - Any MUAC regression - 3 consecutive weeks with no weight gain - Any weight regression | | - Failed appetite test - Any MUAC regression - 3 consecutive weeks with no weight gain - Any weight regression |
| **Routine follow-up** | - A weekly CHV visit for MUAC and weight measurement and RUTF collection as per the dosage scale. - A maximum of 8 weekly visits if the child is progressing well. | | - Bi-weekly visits (every 2 weeks) for MUAC measurement and collection of RUSF (1 sachet per day for 16 weeks. - Maximum of 8 visits if the child is progressing well. |
| **Follow-up of referrals** | Some referrals were followed up by the CHVs through home visits but there was no feedback mechanism in place from the health facilities. | | |
| **Where were children being referred to and estimated average distance to referral location** | Children were being referred from the CHV (or the caregiver/child’s home) to the health facilities. Distance approximately 10km or more. | | |
| **Description of dosage schedule and how children move from SAM to MAM or MAM to full recovery** | RUTF based on weight for 7 days | | 1 sachet of RUSF per day for 14 days  Children admitted as MAM were treated according to the MAM protocol until discharge |
|  | Child’s weight (kg) | # Sachets/day |  |
|  | 4.0-6.9 | 2 |  |
|  | 7.0-9.4 | 3 |  |
|  | 9.5-11.9 | 4 |  |
|  | ≥ 12.0 | 5 |  |
|  | Children admitted as SAM were treated according to the SAM protocol until discharge | |  |
| **Medications provided on which weeks** | Week 1: Amoxicillin  Week 2: Albendazole | | Week 2: Albendazole |
| **Home visit protocol** | CHVs conducted home visits in accordance with the iCCM policy; CHVs were expected to visit 15-20 households per month | | |
| **How treatment protocol differed from national CMAM guidelines** | - MUAC-only protocol (admission, discharge); oedema not treated - RUTF provided to children admitted as SAM according to weight through recovery - No transition ration - Additional referral triggers | | |
| **Other notes** | - If a child was admitted for SAM or MAM, s/he received treatment based on the protocol established for SAM or MAM until discharge - A weight tracking procedure was added as a safety protocol; children who did not gain weight or who had lost weight were referred - If a child presented with diarrhea <14 days and SAM, the CHV was instructed to consult with their supervisor on whether or not to proceed with SAM treatment or to refer - If child presented with SAM or MAM and fast breathing, indicating pneumonia, CHVs gave pre-referral treatment and referred the child to the health facility | | |

**Appendix Table 2: Effects of treatment of acute malnutrition by community health volunteers (meta-analysis approach)**

|  | **Isiolo** |  | **Loima** |  | **Total** |
| --- | --- | --- | --- | --- | --- |
| **Recovered** |  |  |  |  |  |
| Control, % | 24 (50.4 ) |  | 61 (50.1 ) |  | 85 (50.2 ) |
| Intervention, % | 76 (79.9 ) |  | 78 (67.5 ) |  | 154 (73.4 ) |
| RD (95% CI) | 30.9% (11.8 to 50.0) |  | 19.0% (5.7 to 32.3) |  | 23.6% (12.5 to 34.7) |
| *% I^2^* | 28.7 |  | 16.3 |  | 23.3 |
| RR (95% CI) | 1.53 (1.14 to 2.05) |  | 1.34 (1.04 to 1.73) |  | 1.40 (1.16 to 1.70) |
| *% I^2^* | 0.0 |  | 26.5 |  | 15.6 |
| **Defaulted** |  |  |  |  |  |
| Control, % | 16 (30.8 ) |  | 31 (26.7 ) |  | 47 (28.0 ) |
| Intervention, % | 8 (8.3 ) |  | 11 (9.7 ) |  | 19 (9.1 ) |
| RD (95% CI) | -24.9% (-45.1 to -4.6) |  | -21.4% (-36.8 to -6.1) |  | -22.5% (-34.0 to -11.0) |
| *I^2^* | 53.6 |  | 68.8 |  | 59.7 |
| RR (95% CI) | 0.35 (0.13 to 0.93) |  | 0.40 (0.12 to 1.34) |  | 0.39 (0.20 to 0.77) |
| *I^2^* | 0.0 |  | 41.1 |  | 5.1 |
| **Nonresponse*** |  |  |  |  |  |
| Control, % | 9 (18.9 ) |  | 27 (22.3 ) |  | 36 (21.2 ) |
| Intervention, % | 11 (11.7 ) |  | 26 (22.8 ) |  | 37 (17.5 ) |
| RD (95% CI) | -2.4% (-19.5 to 14.7) |  | -2.5% (-15.5 to 7.5) |  | -1.7% (-10.7 to 7.2) |
| *I^2^* | 59.6 |  | 16.7 |  | 38.8 |
| RR (95% CI) | 0.49 (0.17 to 1.45) |  | 1.05 (0.61 to 1.80) |  | 0.89 (0.56 to 1.42) |
| *I^2^* | 14.8 |  | 8.3 |  | 4.7 |
| **Length of stay, days**** |  |  |  |  |  |
| Control, mean ± sd | 76.3 ± 31.0 |  | 52.8 ± 27.9 |  | 58.2 ± 26.5 |
| Intervention, mean ± sd | 50.0 ± 25.9 |  | 49.8 ± 26.1 |  | 50.6 ± 22.9 |
| MD (95% CI) | -23.2 (-43.5 to -2.9) |  | -4.1 (-15.4 to 7.2) |  | -11.9 (-24.5 to 0.6) |
| *I^2^* | 51.5 |  | 21.8 |  | 56.8 |
| **Weight gain, g/kg/day**** |  |  |  |  |  |
| Control, mean ± sd | 0.6 ± 0.9 |  | 1.9 ± 4.1 |  | 1.7 ± 2.1 |
| Intervention, mean ± sd | 1.3 ± 3.5 |  | 2.9 ± 5.8 |  | 2.1 ± 4.3 |
| MD (95% CI) | 0.8 (-0.2 to 1.7) |  | 1.1 (0.2 to 2.0) |  | 1.0 (0.3 to 1.6) |
| *I^2^* | 0.0 |  | 0.0 |  | 0.0 |

* In Isiolo, one cluster pair was dropped in estimating the risk ratio because no child had the outcome.

**Among the recovered children. RD: Risk difference; RR: Risk ratio; MD: Mean difference. This table presents the results of meta-analysis of cluster-level effects to account for few clusters.

**Appendix Table 3: Impacts of treatment of acute malnutrition by community health volunteers (sensitivity analysis –Lee’s bounds)**

|  | **Isiolo** |  | **Loima** |  | **Total** |
| --- | --- | --- | --- | --- | --- |
| **Recovery** |  |  |  |  |  |
| Control, n (%) | 52 (67.5) |  | 86 (59.3) |  | 138 (62.2) |
| Intervention, n (%) | 76 (80.0) |  | 78 (67.8) |  | 154 (73.3) |
| RD (95% CI) -- Upper | 49.5 (27.9, 71.1) |  | 31.8 (15.6, 47.9) |  | 40.7 (25.4, 54.9) |
| RR (95% CI) -- Upper | 2.52 (1.39, 4.56) |  | 1.79 (1.27, 2.54) |  | 2.12 (1.51, 2.98) |
| RD (95% CI) -- Lower | 0 (-14.7, 14.6) |  | 3.7 (-8.8, 16.1) |  | 4.3 (-7.5, 16.0) |
| RR (95% CI) -- Lower | 0.99 (0.81, 1.23) |  | 1.06 (0.86, 1.31) |  | 1.07 (0.89, 1.28) |
|  |  |  |  |  |  |
| **Default*** |  |  |  |  |  |
| Control, n (%) | 44 (57.1) |  | 56 (38.6) |  | 100 (45.1) |
| Intervention, n (%) | 8 (8.4) |  | 11 (9.6) |  | 19 (9.0) |
| RD (95% CI) -- Upper | 0.0 (-9.3,9.3) |  | -4.6 (-14.5, 5.3) |  | -3.1 (-10.4, 4.1) |
| RR (95% CI) -- Upper | 1.00 (0.61, 1.64) |  | 0.79 (0.51, 1.24) |  | 0.85 (0.59, 1.23) |
| RD (95% CI) -- Lower | -50.5 (-76.7, -24.2) |  | -32.9 (-47.4, -18.6) |  | -39.8 (-55.7, 23.9) |
| RR (95% CI) -- Lower | 0.12 (0.05,0.29) |  | 0.19 (0.11, 0.35) |  | 0.16 (0.09, 0.28) |
|  |  |  |  |  |  |
| **Nonresponse*** |  |  |  |  |  |
| Control, n (%) | 37 (48.1) |  | 52 (35.9) |  | 89 (40.1) |
| Intervention, n (%) | 11 (11.6) |  | 26 (22.6) |  | 37 (17.6) |
| % RD (95% CI) -- Upper | 11.8 (-1.8, 25.2) |  | 10.0 (-1.8, 21.8) |  | 9.1 (-1.3, 19.5) |
| RR (95% CI) -- Upper | 2.06 (0.90,4.70) |  | 1.53 (0.89, 2.61) |  | 1.56 (0.91, 2.65) |
| RD (95% CI) -- Lower | -37.4 (-55.6, -19.2) |  | -17.5 (-31.5, -3.5) |  | -27.2 (-39.7, -14.6) |
| RR (95% CI) -- Lower | 0.22 (0.11, 0.44) |  | 0.53 (0.32, 0.87) |  | 0.36 (9.22, 0.57) |
|  |  |  |  |  |  |
| **Length of stay, days**** |  |  |  |  |  |
| Control, mean ± sd | 72.7 ± 29.4 |  | 52.6 ± 23.1 |  | 58.2 ± 26.5 |
| Intervention, mean ± sd | 50.5 ± 23.4 |  | 50.7 ± 22.7 |  | 50.6 ± 22.9 |
| MD (95% CI) ---Upper | 5.34 (-26.3, 37.0) |  | 29.86 (14.73, 44.99) |  | 21.13 (4.81, 37.45) |
| MD (95% CI) --- Lower | -61.13 (-79.3, -42.9) |  | -42.22 (-59.33, -25.10) |  | -51.25 (-65.31, -37.19) |
|  |  |  |  |  |  |
| **Weight gain, g/kg/day **** |  |  |  |  |  |
| Control, mean ± sd | 0.8 ± 1.0 |  | 2.0 ± 3.1 |  | 1.7 ± 2.1 |
| Intervention, mean ± sd | 1.4 ± 3.2 |  | 2.7 ± 5.1 |  | 2.1 ± 4.3 |
| MD (95% CI) ---Upper | 12.0 (5.14, 18.92) |  | 13.67 (9.15, 18.18) |  | 13.33 (9.72, 16.94) |
| MD (95% CI) --- Lower | -21.28 (-33.92, -8.65) |  | -19.44 (-24.84, -14.04) |  | -20.39 (-26.53, -14.25) |

* In Isiolo, one cluster pair was dropped in estimating the risk ratio because no child had the outcome

**Among the recovered children. RD: Risk difference; RR: Risk ratio; MD: Mean difference. The Lee’s bounds are used to account for missing values.
